# *LOXL1‐AS1* communicating with TIAR modulates vasculogenic mimicry in glioma via regulation of the *miR‐374b‐5p/*MMP14 axis

**DOI:** 10.1111/jcmm.17106

**Published:** 2021-12-08

**Authors:** Bolong Yi, Hao Li, Heng Cai, Xin Lou, Mingjun Yu, Zhen Li

**Affiliations:** ^1^ Department of Neurosurgery Shengjing Hospital of China Medical University Shenyang China; ^2^ Liaoning Clinical Medical Research Center in Nervous System Disease Shenyang China; ^3^ Key Laboratory of Neuro‐oncology in Liaoning Province Shenyang China; ^4^ Department of Anesthesiology General Hospital of Shenyang Commend Shenyang

**Keywords:** glioma, *LOXL1‐AS1*, *miR‐374b‐5p*, MMP14, TIAR, vasculogenic mimicry

## Abstract

At present, growing evidence indicates that long non‐coding RNAs (lncRNAs) participate in the progression of glioma. The function of *LOXL1*‐*AS1* in vasculogenic mimicry (VM) in glioma remains unclear. First, the expressions of TIAR, the lncRNA *LOXL1*‐*AS1*, *miR*‐*374b*‐*5p* and MMP14 were examined by qRT‐PCR and Western blot in both, glioma tissues and glioma cell lines. Proliferation, migration, invasion and tube formation assays were conducted to evaluate the roles of TIAR, *LOXL1*‐*AS1*, *miR*‐*374b*‐*5p* and MMP14 in malignant cellular behaviours in glioma cells. A nude mouse xenograft model and dual staining for CD34 and PAS were used to assess whether VM was affected by TIAR, *LOXL1*‐*AS1* or *miR*‐*374b*‐*5p* in vivo. In this study, low levels of TIAR and high levels of *LOXL1*‐*AS1* were found in glioma cells and tissues. TIAR downregulated the expression of *LOXL1*‐*AS1* by destabilizing it. *LOXL1*‐*AS1* acted like a miRNA sponge towards *miR*‐*374b*‐*5p* so that downregulation of the former greatly inhibited cell proliferation, migration, invasion and VM. Additionally, *miR*‐*374b*‐*5p* overexpression repressed malignant biological behaviours and VM in glioma by modifying MMP14. In summary, we demonstrated that TIAR combined with *LOXL1*‐*AS1* modulates VM in glioma via the *miR*‐*374b*‐*5p*/MMP14 axis, revealing novel targets for glioma therapy.

## 1 INTRODUCTION

Glioma is commonly acknowledged as one of the most malignant tumours of the central nervous system and is characterized by high mortality and low survival rates.^1^ Despite the development of various surgeries and medicines over the years, the prognosis of glioma patients remains poor.^2^, ^3^, ^4^ Therefore, exploring new targets for glioma therapy should be prioritized.

Currently, anti‐angiogenesis therapy is becoming increasingly attractive to surgeons worldwide. However, for some reason, the prognosis has not improved much with anti‐vascular endothelial cell therapy. Vasculogenic mimicry (VM), a newly discovered form of angiogenesis in which vessels are surrounded by tumour cells rather than vascular endothelial cells, is now gaining attention.^5^ Several reports have discovered VM in diverse cancers, such as glioma, hepatocellular carcinoma, gastric cancer, non–small‐cell lung cancer and colorectal cancer.^6^, ^7^, ^8^, ^9^, ^10^ VM has a close association with tumour metastasis and is a predictor of poor clinical prognosis.^11^, ^12^

RNA‐binding proteins (RBPs) participate in tumour development, playing roles in pre‐mRNA splicing, translation and RNA stabilization.^13^ Increased TIA1‐related protein (TIAR) expression represses the proliferation of 293 cell lines and xenograft tumour growth.^14^ Additionally, TIAR could prolong the survival of patients with astrocytoma and glioblastoma multiforme, while G3BP1 has the opposite effect.^15^ However, the expression of TIAR in glioma and its possible association with progression have not yet been reported.

Long non‐coding RNAs (lncRNAs) are non‐coding RNAs >200 nucleotides in length. Growing evidence shows that lncRNAs play a vital role in oncogenesis and tumour growth. *LOXL1 antisense RNA1* (*LOXL1*‐*AS1*) downregulates *miR*‐*708*‐*5p* and promotes malignant behaviour in breast cancer.^16^ Additionally, *LOXL1*‐*AS1* participates in regulating drug resistance in prostate cancer by modifying *miR*‐*let*‐*7a*‐*5p* and EGFR.^17^ Furthermore, *LOXL1*‐*AS1* exerts an oncogenic role in laryngocarcinoma, ovarian cancer and colorectal cancer.^18^, ^19^, ^20^ Nevertheless, the mechanism of action of *LOXL1*‐*AS1* in glioma, especially in VM, is elusive.

MicroRNAs (miRNAs) participate in the post‐transcriptional processes of tumorigenesis by regulating the 3′‐UTR of downstream target genes. *miR*‐*374b*‐*5p* downregulates ABCA8 and promotes carcinogenesis in hepatocellular carcinoma.^21^ Moreover, *MiR*‐*374b*‐*5p* plays an antitumour role in pancreatic and cervical cancers.^22^, ^23^ Nevertheless, we know little about *miR*‐*374b*‐*5p* expression and its function in VM in gliomas.

Matrix metalloproteinase 14 (MMP14), a member of the membrane‐type MMP family, is strongly associated with tumour metastasis. MMP14 also plays a VM‐related role in gastric carcinoma, hepatocellular carcinoma and lung cancer.^24^, ^25^, ^26^

This study aimed to investigate the expressions of TIAR, *LOXL1*‐*AS1* and *miR*‐*374b*‐*5p* in both, glioma tissues and glioma cells. Further, the functions of TIAR, *LOXL1*‐*AS1*, *miR*‐*374b*‐*5p* and MMP14, and their interactions in modulating cellular behaviours and VM in glioma are yet to be established. Our study provides new potential therapeutic targets for glioma therapy.

## 2 MATERIALS AND METHODS

### 2.1 Clinical specimens

We obtained 37 tissues in total, according to the WHO classification of central nervous system tumours, including 13 low‐grade glioma tissues (LGGTs; WHO I‐II), 16 high‐grade glioma tissues (HGGTs; WHO III‐IV) and 8 normal brain tissues (NBTs). All tissues were from material discarded during surgery for glioma or traumatic brain injury. Ethical approval was obtained for the current research.

### 2.2 Cell culture

Normal human astrocytes (NHA) along with HEK293T, U87 and U251 cell lines were purchased from the Shanghai Cell Bank affiliated to the Chinese Academy of Life Sciences. NHA were incubated in 1640 medium, while the HEK293T, U87 and U251 cells were incubated in high‐glucose medium with 10% foetal bovine serum (Gibco). The growth conditions were 37°C and 5% CO_2_, together with a certain degree of humidity.

### 2.3 Quantitative real‐time PCR (qRT‐PCR)

After the total RNA was extracted according to the manufacturer's manual of Trizol reagent, *TIAR*, *LOXL1*‐*AS1*, MMP14 and GAPDH expressions were determined using SYBR One Step RT‐PCR kits (Takara). The detections of *miR*‐*374b*‐*5p* and *U6* were performed using the miRNA First Strand cDNA Synthesis kit and the MicroRNA qPCR Kit (Sangon Biotech,). All primers were designed by Sangon Biotech and are presented in Table S1.

### 2.4 Western blot analysis

After electrophoresis, the proteins were transferred to polyvinylidene difluoride membranes (0.22 µm). The membranes were immersed in 5% non‐fat milk at room temperature for 2 h and then incubated with primary antibodies at 4°C for 16–18 h. The primary antibodies were as follows: anti‐TIAR (1:500, Proteintech), anti‐MMP14 (1:1000, Proteintech) and anti‐GAPDH (1:10000, Proteintech). Next, the membranes were incubated with secondary antibodies at room temperature for 2 h, and bands were detected using enhanced chemiluminescence (ECL) reagents from the ECL Detection System. The relative expressions of the proteins were calculated based on the internal reference, GAPDH.

### 2.5 Cell transfection

Full‐length plasmid *TIAR* (*TIAR*[+]), *LOXL1*‐*AS1* (*LOXL1*‐*AS1*[+]), *miR*‐*374b*‐*5p* (pre‐*miR*‐*374b*‐*5p*), *MMP14* (*MMP14*[+]) and their negative controls (NC) were constructed by GenePharma (Shanghai, China). The shRNAs against *TIAR* (*TIAR*[−]), *LOXL1*‐*AS1* (*LOXL1*‐*AS1*[−]), *miR*‐*374b*‐*5p* (anti‐*miR*‐*374b*‐*5p*) and *MMP14* (*MMP14*[−]), and their negative controls were constructed by GeneChem. After cell fusion was approximately 70–80%, the cells were transfected in a 24‐well plate with Lipofectamine 3000. To screen for stably expressing transfected cells, we added G418, puromycin or hygromycin B to the medium. The transfection efficiency was determined using qRT‐PCR and Western blotting (Figure S1). To identify the effects of TIAR on biological behaviour and VM in glioma, we divided the cells into five groups: control, *TIAR*(−)NC, *TIAR*(−), *TIAR*(+)NC and *TIAR*(+). To investigate the corresponding effects of *LOXL1*‐*AS1*, we divided the cells into five groups: control, *LOXL1*‐*AS1*(−)NC, *LOXL1*‐*AS1*(−), *LOXL1*‐*AS1*(+)NC and *LOXL1*‐*AS1*(+). To explore the effects of overexpression of TIAR combined with knockdown of *LOXL1*‐*AS1* on malignant cell behaviours and VM, we divided the cells into five groups: control, *TIAR*(+)NC+LOXL1‐*AS1*(−)NC, *TIAR*(+)+*LOXL1*‐*AS1*(−)NC, *TIAR*(+)NC+LOXL1‐*AS1*(−) and *TIAR*(+)+*LOXL1*‐*AS1*(−). To explore whether *LOXL1*‐*AS1* had a rescue effect on TIAR, we divided the cells into three groups: control, *TIAR*(+)+*LOXL1*‐*AS1*(+)NC and *TIAR*(+)+*LOXL1*‐*AS1*(+). To explore the effects of *miR*‐*374b*‐*5p* on malignancy and VM in glioma, we divided the cells into five groups: control, pre‐*NC*, pre‐*miR*‐*374b*‐*5p*, anti‐*NC* and anti‐*miR*‐*374b*‐*5p*. To confirm whether *miR*‐*374b*‐*5p* expression correlates with that of *LOXL1*‐*AS1*, we divided the cells into five groups: control, *LOXL1*‐*AS1*(−)NC+pre‐NC, *LOXL1*‐*AS1*(−)+pre‐*miR*‐*374b*‐*5p*, *LOXL1*‐*AS1*(−)NC+anti‐NC and *LOXL1*‐*AS1*(−)+anti‐*miR*‐*374b*‐*5p*. To identify the mechanism of MMP14 interaction with *miR*‐*374b*‐*5p* in VM, we divided the cells into five groups: control, pre‐NC+MMP14(−)NC, pre‐*miR*‐*374b*‐*5p*+*MMP14*(−), pre‐NC+MMP14(+)NC and pre‐*miR*‐*374b*‐*5p*+*MMP14*(+).

### 2.6 RNA immunoprecipitation (RIP)

Following the manufacturer's protocol, we performed RIP assays in which the whole‐cell lysate was incubated with magnetic beads from the EZ‐Magna RIP kit (Millipore, Billerica, MA). The beads were conjugated with anti‐Ago 2 antibody or normal mouse IgG. After incubation in proteinase K buffer, the immunoprecipitated RNA was extracted and analysed by qRT‐PCR.

### 2.7 Fluorescence in situ hybridization (FISH)

After blocking in prehybridization buffer, slides were disposed with PCR‐grade proteinase‐k (Roche Diagnostics, Germany). *LOXL1*‐*AS1* probe (GenePharma), which was constructed to confirm the localization of *LOXL1*‐*AS1* in glioma cells, was added to the hybridization solution. Afterwards, the sections were stained with anti‐digoxin rhodamine conjugate (Exon Biotech Inc.,) at 37°C for 1 h and then DAPI (Beyotime) for 2 min. All images were captured under a fluorescence microscope.

### 2.8 Dual‐luciferase reporter assays

To investigate the interplay of *LOXL1*‐*AS1* and *miR*‐*374b*‐*5p*, HEK293T cells were co‐transfected with *LOXL1*‐*AS1*‐Wt or *LOXL1*‐*AS1*‐Mut vector after seeding in a 96‐well plate, and the effect of overexpression of *miR*‐*374b*‐*5p* vs. NC was assessed. To investigate the interplay of *miR*‐*374b*‐*5p* and MMP14, HEK293T cells were co‐transfected with *MMP14*‐3′UTR‐Wt or *MMP14*‐3′UTR‐Mut vector, and the overexpression of *miR*‐*374b*‐*5p* vs. NC was assessed. After 48 h, the relative luciferase activity was calculated and analysed using the Dual‐Luciferase Reporter Assay System (Promega).

### 2.9 Nascent RNA assay

Per the manufacturer's protocol, the expression of nascent *LOXL1*‐*AS1* was examined using the Click‐iT Nascent RNA Capture Kit (Life Technologies Corporation). In general, we used 0.2 mM of ethylene uridine (EU) ribonucleotide homologs to mark the nascent RNA, and the RNA so labelled was released from the magnetic beads and collected. Finally, the nascent RNA expression was determined using qRT‐PCR.

### 2.10 RNA stability assay

After transfection with *TIAR*(+) and its NC, HEK293 cells were treated with Actinomycin D (5 mg/ml, Sigma‐Aldrich), and the total RNA was obtained in real time and detected using qRT‐PCR.

### 2.11 Cell proliferation assay

To assess the cell viability, cells were resuspended and seeded into a 96‐well plate. The Cell Counting Kit‐8 reagent was used per the instructions of the manufacturer. The cell viability was determined using a microplate reader.

### 2.12 Cell migration and invasion assays

*Migration assay*: After re‐suspension in a serum‐free medium, approximately 1 × 10^5^ U87 or U251 cells were seeded into a transwell. Simultaneously, the cell‐culture medium was added to the side beneath the transwell chamber. Next, the cells were fixed and stained with 10% Giemsa in phosphate buffer at room temperature overnight. Then, cell numbers were counted in three random fields under a microscope. *Modification for invasion assay*: We coated 70 μl of Matrigel solution (Corning) with a density of 50 mg/ml on the upper side of the transwell before seeding the cells.

### 2.13 Tube formation assay

The 3D model tube was formed using a gel. After U87 and U251 cells were successfully transfected, we resuspended the cells and seeded them into a 24‐well plate pre‐coated with Matrigel. Tube formation was then observed and analysed in three random fields under a microscope immediately after incubation at 37°C for nearly 6 h.

### 2.14 CD34 and PAS dual staining

Tissue specimens were fixed in 4% paraformaldehyde and embedded in paraffin, microtomed, and de‐paraffinized in xylene and graded ethanol solutions. Specimens were then placed in a citrate antigen retrieval solution and heated in a microwave oven at a temperature controlled at close to the boiling point. After subsequent incubation with 3% hydrogen peroxide and goat serum, the specimens were incubated in CD34 primary antibody at a ratio of 1:100 at 4°C overnight. Subsequently, the specimens were incubated with the secondary antibody at room temperature for 20 min and stained with a DAB kit immediately. The PAS reaction was performed using periodic acid and Schiff staining reagents. The specimens were then permanently preserved with neutral resins and stained with haematoxylin. The number of VM tubes was counted and analysed using a normal microscope.

### 2.15 Xenograft tumour in nude mice

Glioma cells were transfected and screened for stable expression. An in vivo xenograft tumour model was then established in nude mice using stably expressing transfected cells. BALB/C athymic nude mice aged 4 weeks old with a weight of 14–16 g (Beijing HUAFUKANG bioscience) were divided into five groups: control, *TIAR*(+), *LOXL1*‐*AS1*(−), pre‐*miR*‐*374b*‐*5p* and *TIAR*(+)+*LOXL1*‐*AS1*(−)+pre‐*miR*‐*374b*‐*5p*. Each group contained ten mice to ensure experimental accuracy. After transfection and re‐suspension at a density of 2 × 10^6^/ml, 100 ml of cell suspension was injected subcutaneously into each mouse. The volume of tumours was measured and calculated every four days using the following formula: volume (mm^3^) = length × width^2^/2. All mice were sacrificed after 44 days. We implanted 1 × 10^5^ cells into the right striatum of nude mice in the orthotopic inoculation experiments. Survival time was analysed using the Kaplan‐Meier survival curve.

Importantly, this research was approved by a panel of experts on laboratory animal care and was conducted following the standards for the care and handling of laboratory animals.

### 2.16 Statistical analysis

All experimental data shown as mean ± standard deviation were analysed using SPSS 22.0. Statistical significance was set at *p* < 0.05.

## 3 RESULTS

### 3.1 TIAR shows low expression levels in glioma tissues and cells, and its overexpression represses VM by glioma cells

**FIGURE 1:**
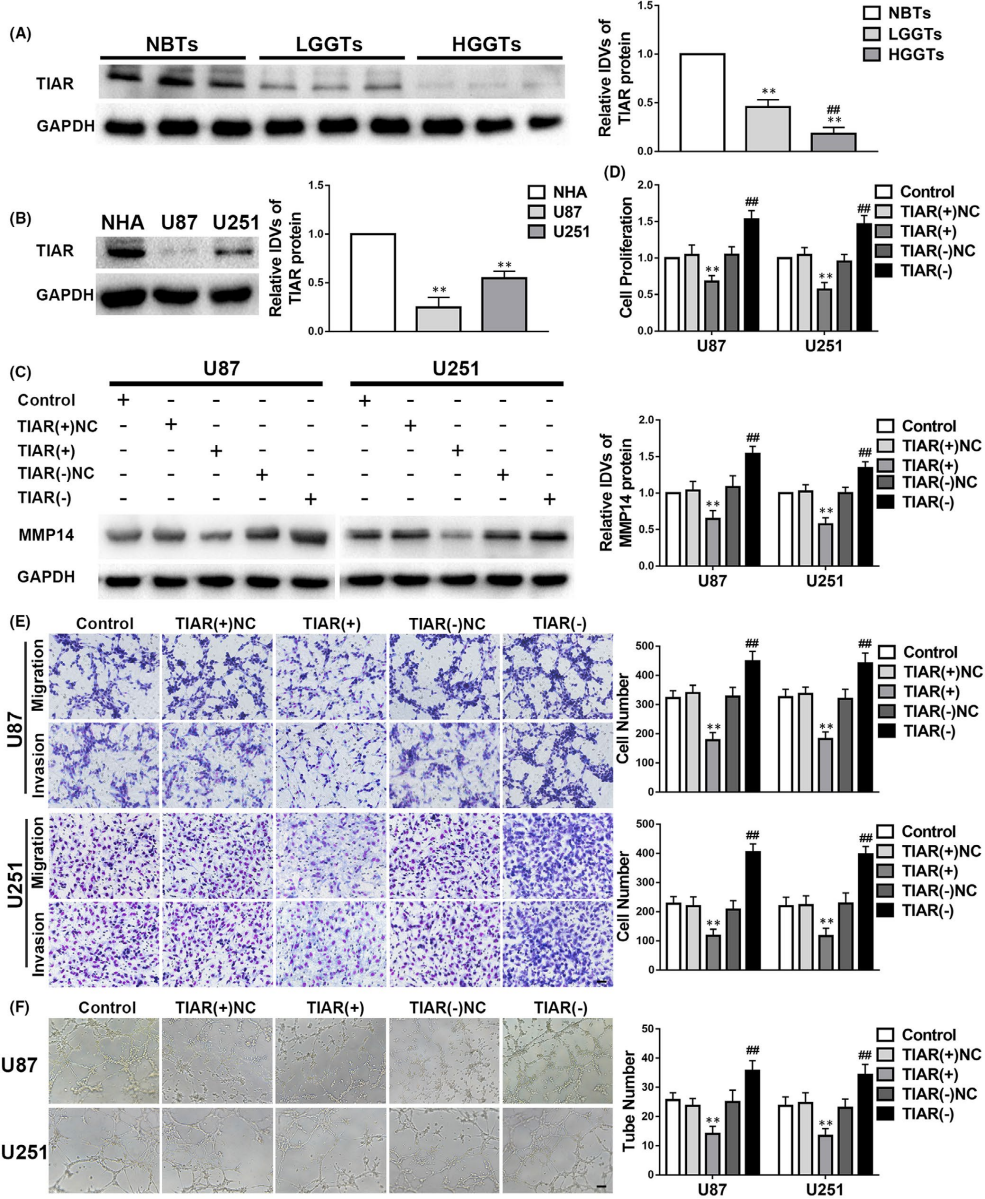
TIA1‐related protein (TIAR) plays a suppressive role in glioma. A–F. The data are displayed as mean ± standard deviation. A. The TIAR expression in glioma tissues is shown. *n* = 8, normal brain tissues (NBTs); *n* = 13, low‐grade glioma tissues (LGGTs); *n* = 18, high‐grade glioma tissues (HGGTs); **p < 0.01 vs. NBTs; ##*p* < 0.01 vs. LGGTs. B. TIAR expression in glioma cells. *n* = 5, each group; **p < 0.01 vs. NHA. C. MMP14 expression is regulated by TIAR. *n* = 5, each group; negative controls (NC); **p < 0.01 vs. TIAR(+)NC; ##*p* < 0.01 vs. TIAR(−)NC. D–F. Proliferation, migration, invasion, and vasculogenic mimicry (VM) are regulated by TIAR. *n* = 3, each group; **p < 0.01 vs. TIAR(+)NC; ##*p* < 0.01 vs. TIAR(−)NC. Scale bars indicate 100 μm in the migration and invasion assays and 200 μm in the tube formation test of VM

To detect TIAR expression, Western blot analysis was performed. The results demonstrated that TIAR expression was lower in glioma tissues and cells than those in NBTs (Figure 1A) and NHA cells (Figure 1B). To investigate the role of TIAR in glioma, we evaluated the effects of TIAR overexpression and knockdown on cell proliferation, migration, invasion and VM. We observed that MMP14 was downregulated in the *TIAR*(+) group and upregulated in the *TIAR*(−) group in comparison with the corresponding NC group (Figure 1C). Cell proliferation, migration, invasion and VM were repressed in the *TIAR*(+) group but elevated in the *TIAR*(−) group compared to their NC groups (Figure 1D–F).

### 3.2 *LOXL1*‐*AS1* is upregulated in glioma tissues and cells, and its knockdown suppresses VM in glioma cells

**FIGURE 2:**
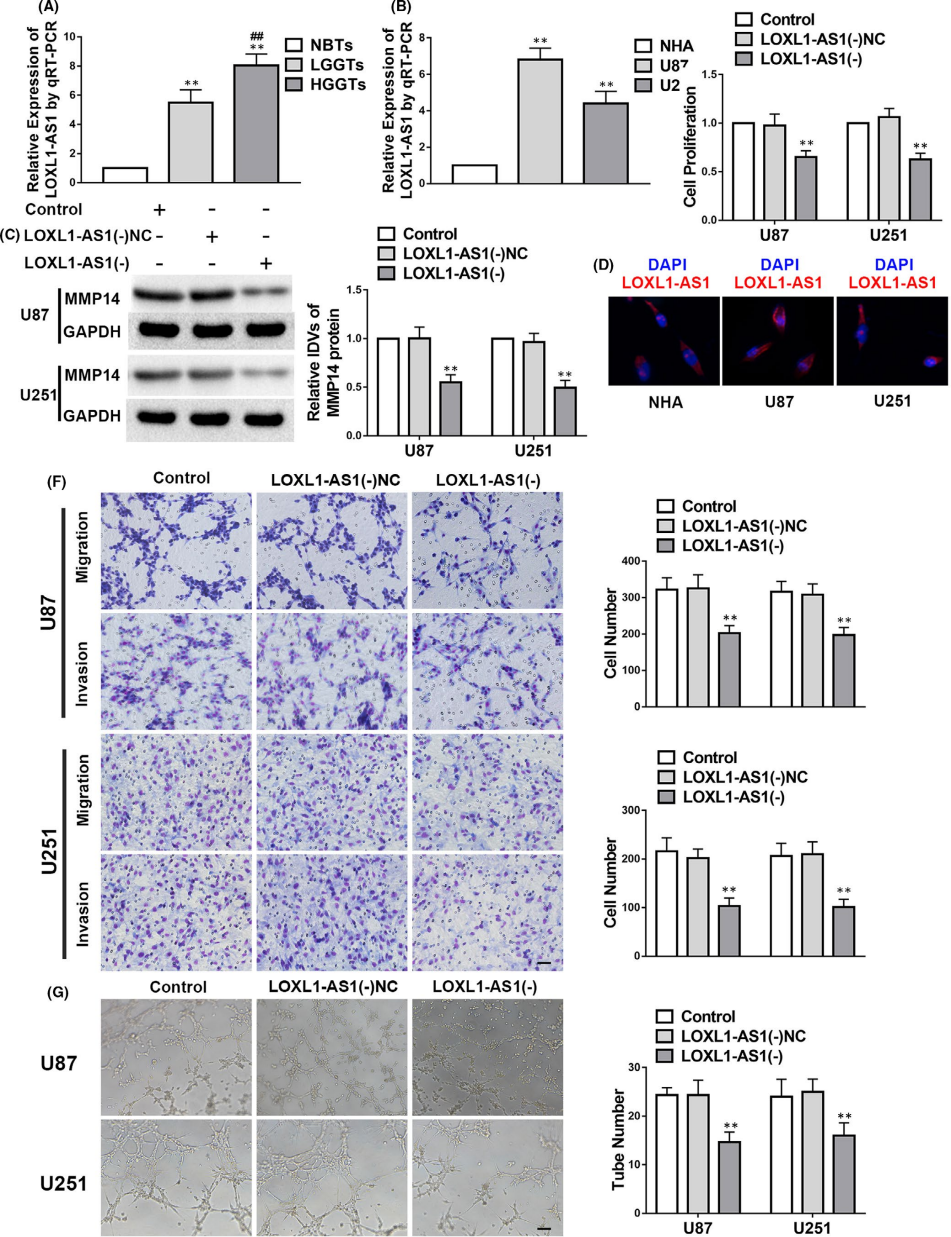
*LOXL1*‐*AS1* functions as an oncogene in glioma. A–G. The data are displayed as mean ± standard deviation. A. *LOXL1*‐*AS1* expression in glioma tissues; *n* = 8, normal brain tissues (NBTs); *n* = 13, low‐grade glioma tissues (LGGTs); *n* = 18, high‐grade glioma tissues (HGGTs); **p < 0.01 vs. NBTs; ##*p* < 0.01 vs. LGGTs. B. *LOXL1*‐*AS1* expression in glioma cells; *n* = 5, each group; **p < 0.01 vs. normal human astrocytes. C. Matrix metalloproteinase 14 (MMP14) expression is modulated by *LOXL1*‐*AS1*. *n* = 3, each group; negative controls (NC); **p < 0.01 vs. *LOXL1*‐*AS1*(−)NC. D. FISH was performed to examine the location and expression of LOXL1‐AS1 in NHA, U87 and U251. E‐G. Proliferation, migration, invasion and vasculogenic mimicry are regulated by TIAR. *n* = 3, each group; **p < 0.01 vs. *LOXL1*‐*AS1*(−)NC. Scale bars indicate 100 μm in the migration and invasion assays and 200 μm in the tube formation assay

*LOXL1*‐*AS1* expression was higher in glioma tissues and cells than in NBTs and NHA, and the higher the grade of glioma, the higher the expression of *LOXL1*‐*AS1* (Figure 2A,B). The FISH experiment confirmed that *LOXL1*‐*AS1* was located in the cytoplasm of the NHA and glioma cells (Figure 2D). To explore the mechanism of action of *LOXL1*‐*AS1* in glioma, experiments involving cell proliferation, migration, invasion and VM were conducted shortly after *LOXL1*‐*AS1* was downregulated. We found that the expression of MMP14 in the *LOXL1*‐*AS1*(−) group was downregulated compared with that in the *LOXL1*‐*AS1*(−)NC group (Figure 2C). Furthermore, as shown in Figure 2E–G, cell proliferation, invasion, migration and VM in the *LOXL1*‐*AS1*(−) group were strikingly suppressed in comparison with those in the *LOXL1*‐*AS1*(−)NC group.

### 3.3 TIAR reduces *LOXL1*‐*AS1* expression by destabilization, and TIAR overexpression and *LOXL1*‐*AS1* knockdown both significantly reduce VM by glioma cells

**FIGURE 3:**
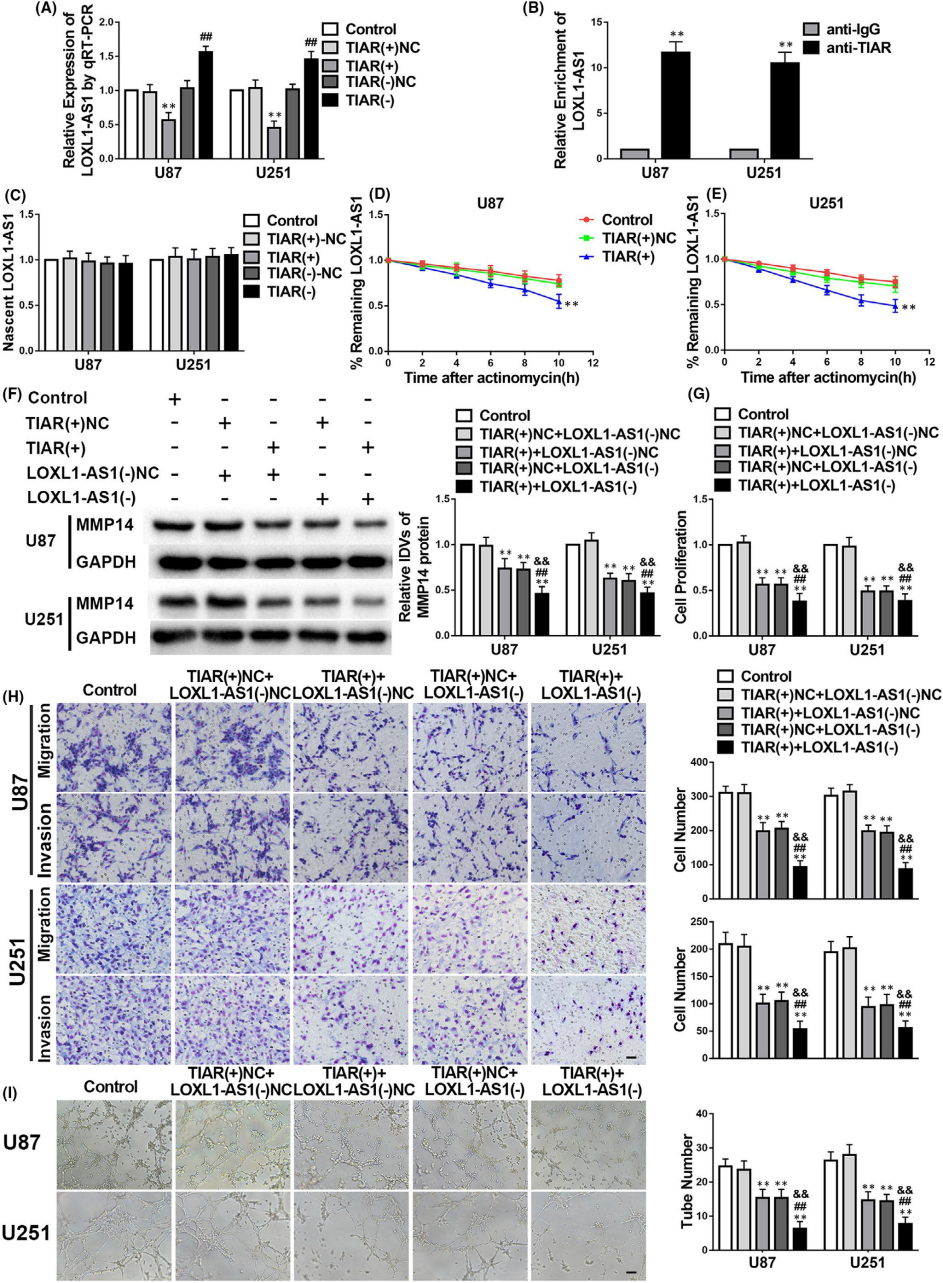
TIA1‐related protein (TIAR) negatively modulates the stability of *LOXL1*‐*AS1* in glioma. A–I. The data are displayed as mean ± standard deviation (*n* = 3, each group). A. *LOXL1*‐*AS1* expression is regulated by TIAR. negative controls (NC); **p < 0.01 vs. TIAR(+)NC; ##*p* < 0.01 vs. TIAR(‐)NC. B. *LOXL1*‐*AS1* forms a complex with TIAR. **p < 0.01 vs. anti‐IgG. C–D. The expression levels of the remaining *LOXL1*‐*AS1* in glioma cells are shown a standard time after treatment with actinomycin D. **p < 0.01 vs. TIAR(+)NC. E. Nascent *LOXL1*‐*AS1* expression is shown. F. The protein level of matrix metalloproteinase 14 (MMP14) is regulated by the co‐transfection of TIAR(+) and LOXL1‐*AS1*(−). negative controls (NC); **p < 0.01 vs. *TIAR*(+)NC+LOXL1‐*AS1*(−)NC; ##*p* < 0.01 vs. *TIAR*(+)+LOXL1‐*AS1*(−)NC; &&*p* < 0.01 vs. *TIAR*(+)NC+LOXL1‐*AS1*(−). G–I. The levels of proliferation, migration, invasion and vasculogenic mimicry in glioma cells confirm the effects of co‐transfection of *TIAR*(+) and LOXL1‐*AS1*(−). **p < 0.01 vs. *TIAR*(+)NC+LOXL1‐*AS1*(−)NC; ##*p* < 0.01 vs. *TIAR*(+)+LOXL1‐*AS1*(−)NC; &&*p* < 0.01 vs. *TIAR*(+)NC+LOXL1‐*AS1*(−). Scale bars indicate 100 μm in the migration and invasion assays and 200 μm in the tube formation assay

Using the Starbase bioinformatics database, we predicted that TIAR binds to *LOXL1*‐*AS1*. Results showed that *LOXL1*‐*AS1* was downregulated in the *TIAR*(+) group but upregulated in the *TIAR*(−) group compared with the respective NC groups (Figure 3A). To further evaluate the correlation between TIAR and *LOXL1*‐*AS1*, TIAR and *LOXL1*‐*AS1* were co‐transfected. As shown in Figure 3F, MMP14 expression was decreased in the *TIAR*(+)+*LOXL1*‐*AS1*(−)NC and *TIAR*(+)NC+LOXL1‐*AS1*(−) groups in comparison with that in the *TIAR*(+)NC+LOXL1‐*AS1*(−)NC group. Furthermore, we found that MMP14 was significantly downregulated in the *TIAR*(+)+*LOXL1*‐*AS1*(−) group. Figure 3B demonstrates a relative enrichment of *LOXL1*‐*AS1* in the RIP experiment as an elevation in the anti‐TIAR group in comparison with the anti‐IgG group. Moreover, nascent RNA and RNA stability experiments showed that nascent *LOXL1*‐*AS1* expression had no statistical difference among the *TIAR*(−), *TIAR*(+), *TIAR*(−)NC and *TIAR*(+)NC groups (Figure 3C). The half‐life of *LOXL1*‐*AS1* was shortened in the *TIAR*(+) group compared with the *TIAR*(+)NC group (Figure 3D,E). Additionally, cell proliferation, migration, invasion and VM were attenuated in the *TIAR*(+)+*LOXL1*‐*AS1*(−)NC and *TIAR*(+)NC+LOXL1‐*AS1*(−) groups and were remarkably reduced in the *TIAR*(+)+*LOXL1*‐*AS1*(−) group in comparison with their respective NC groups (Figure 3G–I).

### 3.4 Overexpression of *LOXL1*‐*AS1* reverses the anti‐cancer effects of TIAR overexpression in glioma

The expression of MMP14 in the *TIAR*(+)+*LOXL1*‐*AS1*(+) group was higher than that in the *TIAR*(+)+*LOXL1*‐*AS1*(+)NC group (Figure S2A). Cell proliferation, migration, invasion and VM in the *TIAR*(+)+*LOXL1*‐*AS1*(+) group were also elevated in comparison with those in the *TIAR*(+)+*LOXL1*‐*AS1*(+)NC group (Figure S2B–D).

### 3.5 *miR*‐*374b*‐*5p* has an anti‐tumour role in glioma, and *miR*‐*374b*‐*5p* overexpression suppresses VM by glioma cells

**FIGURE 4:**
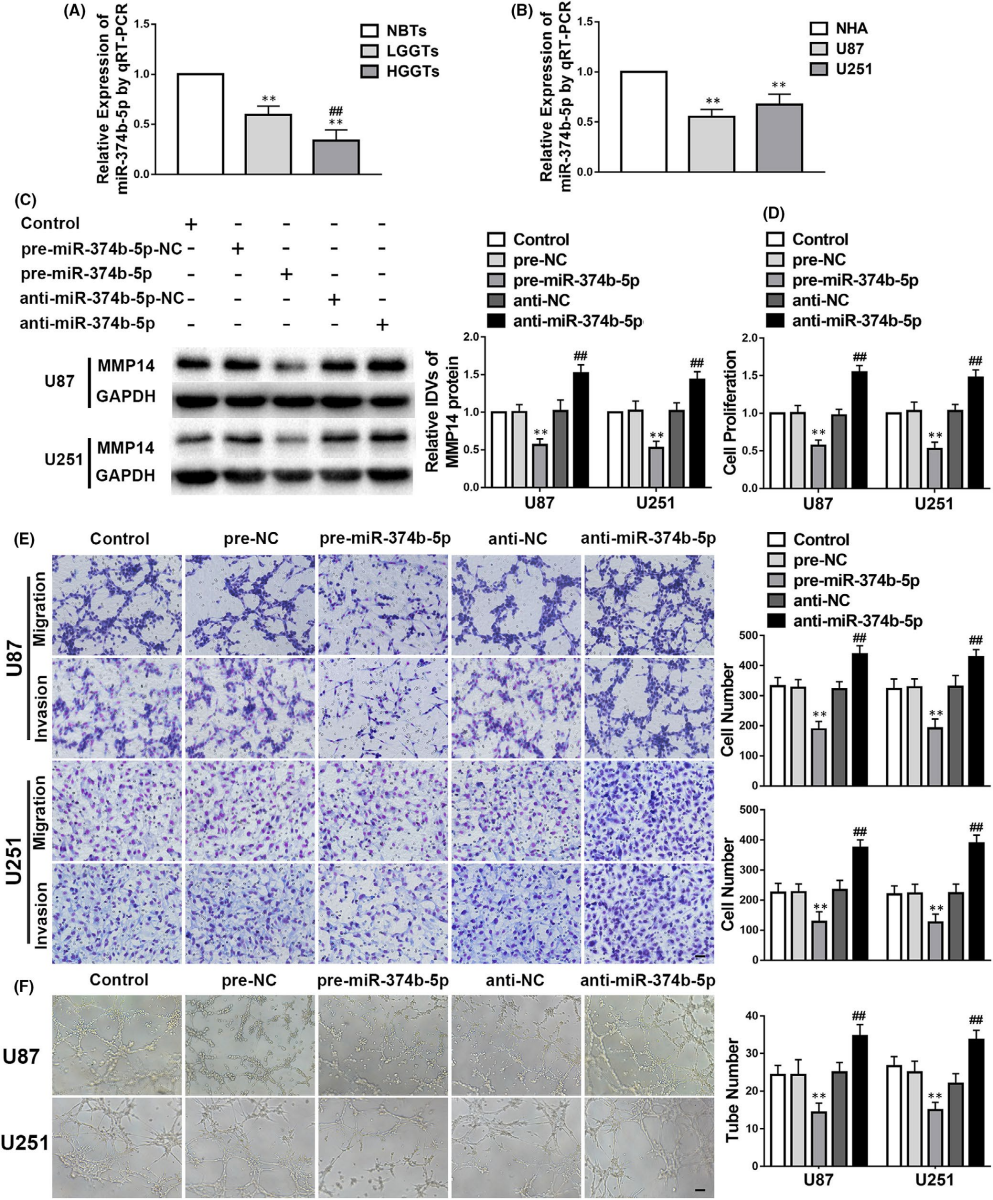
Anti‐tumour effects of *miR*‐*374b*‐*5p* in glioma. A‐F. The data are displayed as mean ± standard deviation. A. *miR*‐*374b*‐*5p* expression in glioma tissues; *n* = 8, normal brain tissues (NBTs); *n* = 13, low‐grade glioma tissues (LGGTs); *n* = 18, high‐grade glioma tissues (HGGTs); **p < 0.01 vs. NBTs; ##*p* < 0.01 vs. LGGTs. B. *miR*‐*374b*‐*5p* expression in glioma cells; *n* = 5, each group; **p < 0.01 vs. normal human astrocytes. C. The expression level of matrix metalloproteinase 14 (MMP14) modulated by *miR*‐*374b*‐*5p*; *n* = 3, each group; negative controls (NC); **p < 0.01 vs. pre‐NC; ##*p* < 0.01 vs. anti‐NC. D–F. Proliferation, migration, invasion, and vasculogenic mimicry regulated by *miR*‐*374b*‐*5p*; *n* = 3, each group; **p < 0.01 vs. pre‐NC; ##*p* < 0.01 vs. anti‐NC. Scale bars indicate 100 μm in the migration and invasion assays and 200 μm in the tube formation assay

*miR‐374b‐5p* was expressed at lower levels in glioma tissues and cells than in NBTs and NHA (Figure 4A,B). To evaluate the function of *miR‐374b‐5p* in glioma, we evaluated the effects of pre‐*miR‐374b‐5p* and anti‐*miR‐374b‐5p* on cell behaviour and VM potential in glioma. As shown in Figure 4C, MMP14 was downregulated in the pre‐*miR‐374b‐5p* group but upregulated in the anti‐*miR‐374b‐5p* group compared with their NC groups. Moreover, cell proliferation, migration, invasion and VM were suppressed in the pre‐*miR‐374b‐5p* group but promoted in the anti‐*miR‐374b‐5p* group in comparison with their NC groups (Figure 4D–F).

### 3.6 *LOXL1*‐*AS1* downregulates *miR*‐*374b*‐*5p*, and *miR*‐*374b*‐*5p* modulates downregulated *LOXL1*‐*AS1* to affect cell behaviour and VM

**FIGURE 5:**
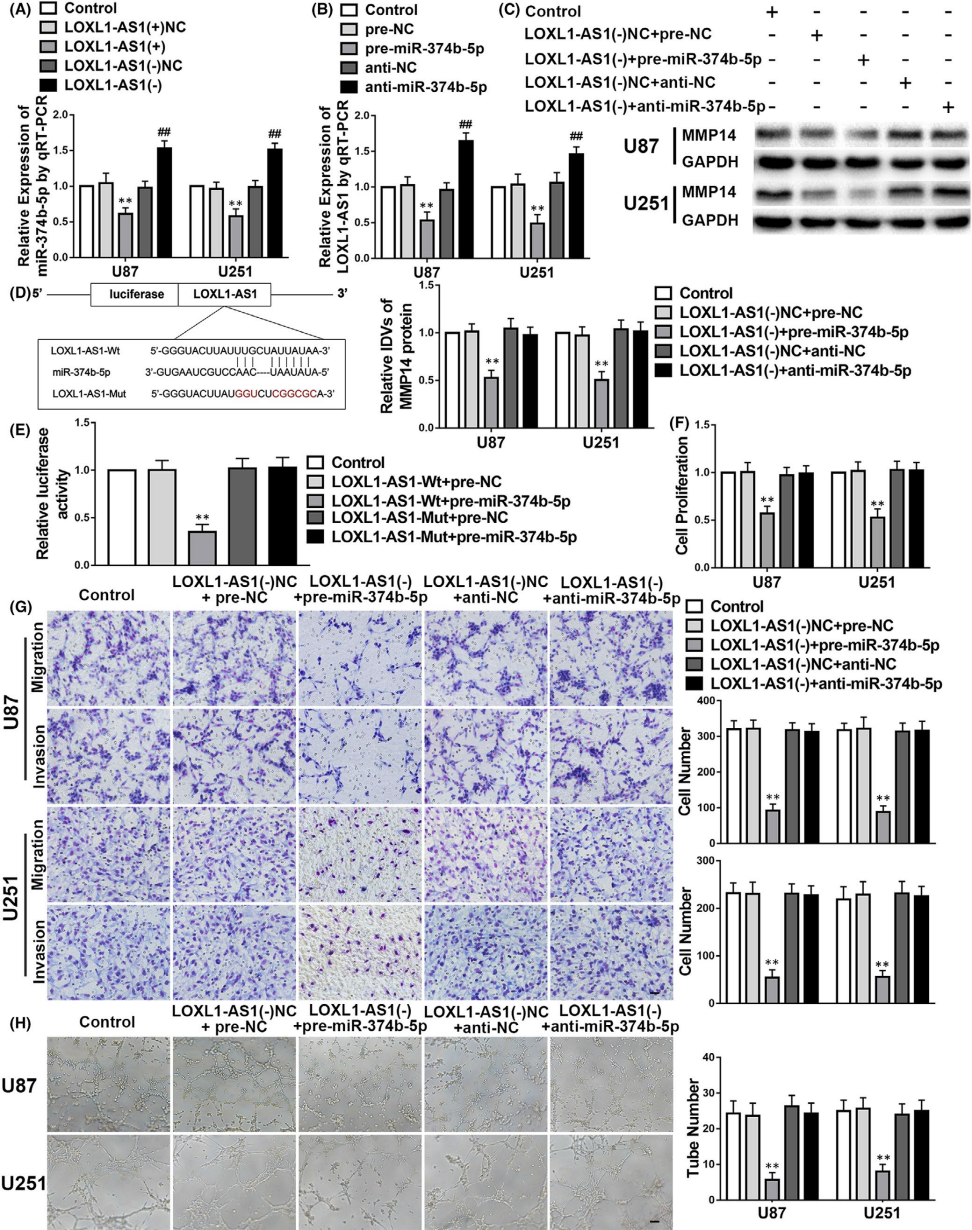
LOXL1‐*AS1* knockdown represses malignant cell behaviours and vasculogenic mimicry (VM) in glioma by upregulating *miR*‐*374b*‐*5p*. A–H. The data are displayed as mean ± standard deviation (*n* = 3, each group). A. *miR*‐*374b*‐*5p* expression is regulated by *LOXL1*‐*AS1*. negative controls (NC); **p < 0.01 vs. *LOXL1*‐*AS1*(+)NC; ##*p* < 0.01 vs. *LOXL1*‐*AS1*(−)NC. B. *LOXL1*‐*AS1* expression is regulated by *miR*‐*374b*‐*5p*. **p < 0.01 vs. pre‐NC; ##*p* < 0.01 vs. anti‐NC. C. The sequence mediating binding between *LOXL1*‐*AS1* and *miR*‐*374b*‐*5p* as well as the mutant sequence is shown. D. The relative luciferase activities after the co‐transfection of pre‐*miR*‐*374b*‐*5p* and *LOXL1*‐*AS1*‐Mut or *LOXL1*‐*AS1*‐Wt are shown. **p < 0.01 vs. *LOXL1*‐*AS1*‐Wt+pre‐NC. E. The expression level of matrix metalloproteinase 14 (MMP14) is regulated by co‐transfection of *LOXL1*‐*AS1* and *miR*‐*374b*‐*5p*. **p < 0.01 vs. *LOXL1*‐*AS1*(−)NC+pre‐NC. F–H. Cellular proliferation, migration, invasion and VM results confirm the co‐operative action of *LOXL1*‐*AS1* and *miR*‐*374b*‐*5p* transfection in glioma. **p < 0.01 vs. *LOXL1*‐*AS1*(−)NC+pre‐NC. Scale bars indicate 100 μm in the migration and invasion assays and 200 μm in the tube formation assay

Initially, we noticed that *miR*‐*374b*‐*5p* was downregulated in the *LOXL1*‐*AS1*(+) group but upregulated in the *LOXL1*‐*AS1*(‐) group compared with the corresponding NCs (Figure 5A). Additionally, *LOXL1*‐*AS1* expression was decreased in the pre‐*miR*‐*374b*‐*5p* group but increased in the anti‐*miR*‐*374b*‐*5p* group in comparison with the pre‐NC and anti‐NC groups respectively (Figure 5B). Figure 5C shows that the MMP14 expression was reduced in the *LOXL1*‐*AS1*(−)+pre‐*miR*‐*374b*‐*5p* group compared with those of their NCs. Furthermore, the dual‐luciferase reporter assay demonstrated that *miR*‐*374b*‐*5p* binds to *LOXL1*‐*AS1* at its 3′‐UTR (Figure 5D,E). To further investigate this interaction, we evaluated the effects of *LOXL1*‐*AS1* and *miR*‐*374b*‐*5p* on cell proliferation, migration, invasion and VM. As shown in Figure 5F–H, cell proliferation, migration, invasion and VM in glioma (henceforth, ‘malignant cell behaviour’) were greatly inhibited in the *LOXL1*‐*AS1*(−)+pre‐*miR*‐*374b*‐*5p* group compared with those of the NCs. We also observed that according to the cell behaviour results, silencing *miR*‐*374b*‐*5p* reversed the tumour‐suppressive function of *LOXL1*‐*AS1*(−)+pre‐*miR*‐*374b*‐*5p*.

### 3.7 MMP14 has an oncogenic role in gliomas and exerts facilitation in VM

As shown in Figure S3A,B, MMP14 was highly expressed in glioma tissues and cells. To figure out its role in gliomas, we assessed the effects of diverse transfections altering MMP14 expressions on the malignant cell behaviour. The malignant cell behaviour was greatly inhibited in the *MMP14*(‐) group but promoted in the *MMP14* (+) group, in comparison with that of their corresponding NCs (Figure S3C–E).

### 3.8 *miR*‐*374b*‐*5p* targets *MMP14* by binding to its 3′UTR; *miR*‐*374b*‐*5p* and MMP14 both regulate malignant cell behaviour

**FIGURE 6:**
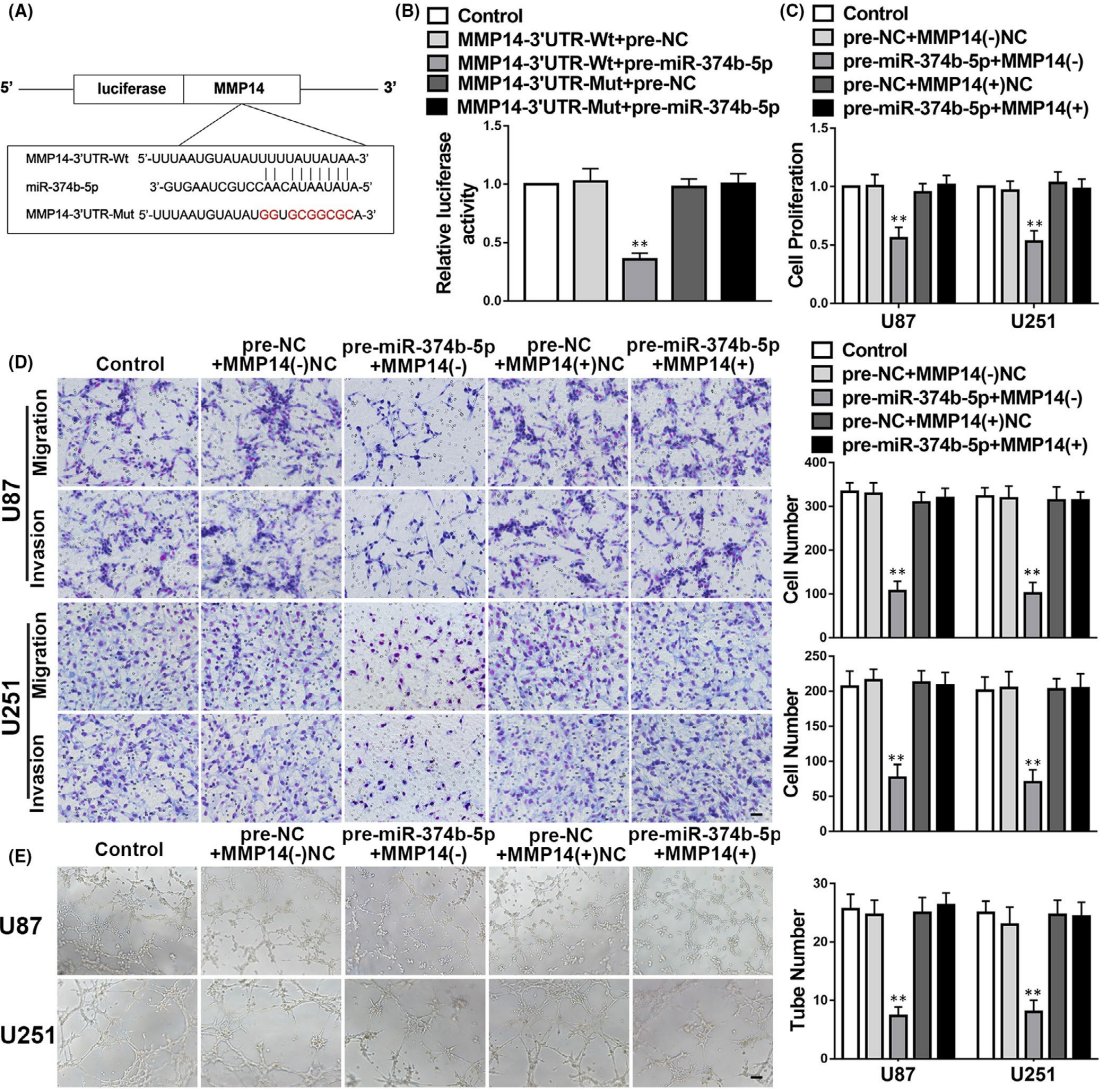
*miR*‐*374b*‐*5p* overexpression represses malignant cellular behaviours and vasculogenic mimicry (VM) in gliomas by targeting MMP14. A. The potential binding sequence for Matrix metalloproteinase 14 (*MMP14)* and *miR*‐*374b*‐*5p* and a mutant sequence is shown. B–E. The data are displayed as mean ± standard deviation (*n* = 3, each group). B. The relative luciferase activities for the co‐transfection of pre‐*miR*‐*374b*‐*5p* and either *MMP14*‐Mut or *MMP14*‐Wt are shown. negative controls (NC); **p < 0.01 vs. *MMP14*‐Wt+pre‐NC. C–E. The cellular proliferation, migration, invasion and VM results confirm the effects of co‐transfection of *MMP14* and *miR*‐*374b*‐*5p* in gliomas. **p < 0.01 vs. *MMP14*(−)NC+pre‐NC. Scale bars indicate 100 μm in the migration and invasion assays and 200 μm in the tube formation assay

The dual‐luciferase reporter assay proved that *miR*‐*374b*‐*5p* binds to *MMP14* at its 3′‐UTR (Figure 6A,B). To explore the role of this interaction, we assessed the effects of diverse transfections altering the expressions of *miR*‐*374b*‐*5p* and MMP14 on malignant cell behaviour. Malignant cell behaviour was greatly inhibited in the pre‐*miR*‐*374b*‐*5p*+*MMP14*(−) group compared to that in their corresponding NCs (Figure 6C–E). Moreover, MMP14 overexpression reversed this effect (Figure 6C–E).

### 3.9 Overexpression of TIAR combined with *LOXL1*‐*AS1* knockdown and overexpression of *miR*‐*374b*‐*5p* inhibits the growth and VM propensity of xenograft tumours in nude mice and prolongs survival

**FIGURE 7:**
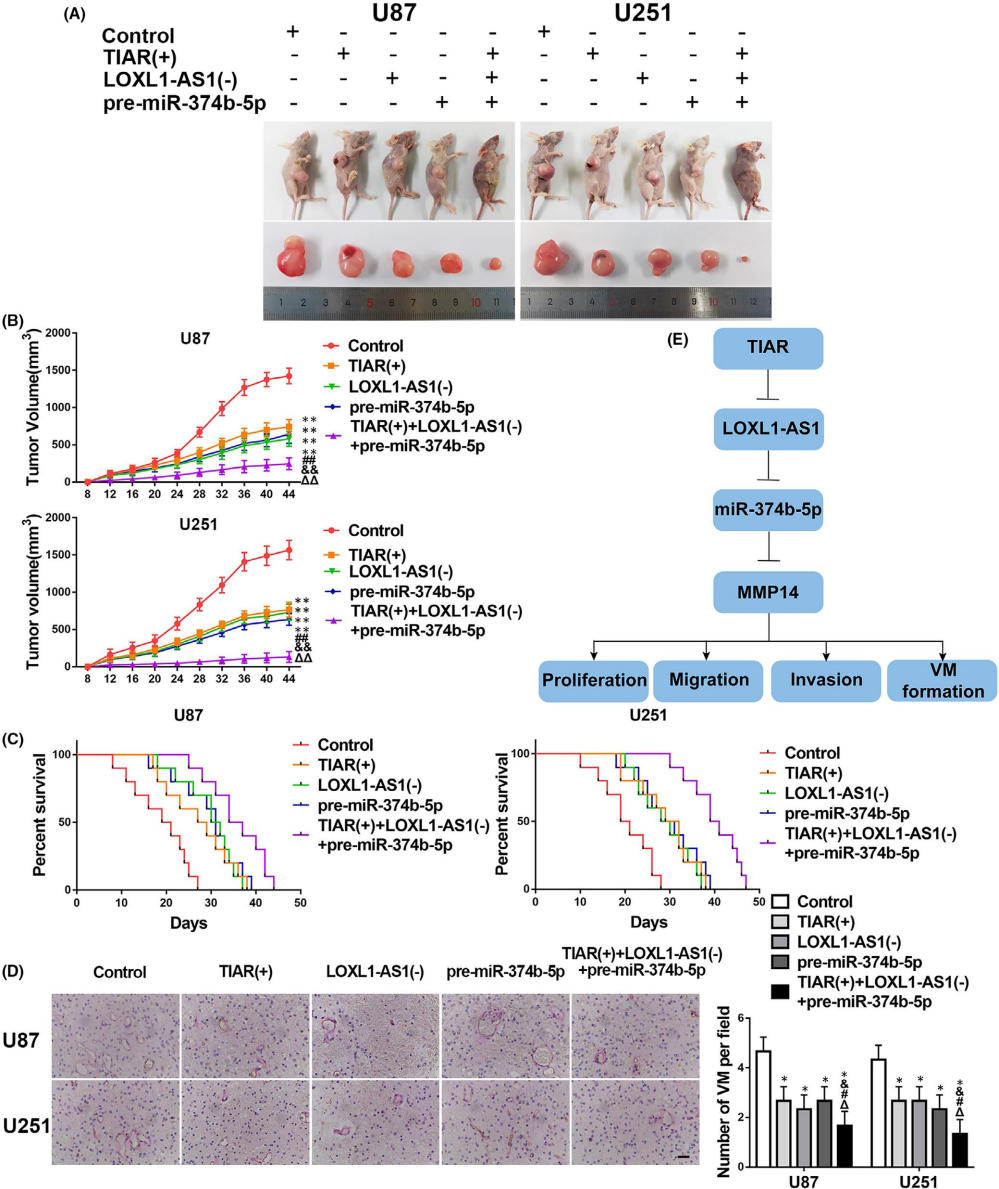
In vivo study. A. The tumours in vivo and the corresponding samples following the subcutaneous implantation of stably expressing cells into nude mice are shown. B. The tumour volumes are shown. The data are displayed as mean ± standard deviation (SD) (*n* = 10, each group). **p < 0.01 vs. control group; ##*p* < 0.01 vs. *TIAR*(+) group; &&*p* < 0.01 vs. *LOXL1*‐*AS1*(−) group; ΔΔp < 0.01 vs. pre‐*miR*‐*374b*‐*5p* group. C. The survival curves of the nude mice groups are shown. *p* < 0.05 (*TIAR*(+), *LOXL1*‐*AS1*(−) and pre‐*miR*‐*374b*‐*5p* vs. control). D. VM in xenograft tumours by CD34‐PAS staining. The data are displayed as mean ± SD (*n* = 5, each group). *p < 0.05 vs. control group; #*p* < 0.05 vs. *TIAR*(+) group; &*p* < 0.05 vs. *LOXL1*‐*AS1*(−) group; Δ*p* < 0.05 vs. pre‐*miR*‐*374b*‐*5p* group. Scale bars indicate 200 μm. E. A schematic of the TIA1‐related protein (TIAR)/*LOXL1*‐*AS1*/*miR*‐*374b*‐*5p* mechanism

A nude mouse xenograft model was established to investigate the functions of TIAR, *LOXL1*‐*AS1* and *miR*‐*374b*‐*5p* in glioma *in vivo*. The results demonstrated that the volume of xenograft tumours was smaller in the *TIAR*(+), *LOXL1*‐*AS1*(−) and pre‐*miR*‐*374b*‐*5p* groups than that in their NCs, and the smallest in the *TIAR*(+)+*LOXL1*‐*AS1*(−)+pre‐*miR*‐*374b*‐*5p* group (Figure 7A,B). The survival time was prolonged in the *TIAR*(+), *LOXL1*‐*AS1*(−) and pre‐*miR*‐*374b*‐*5p* groups compared to that of their NCs (Figure 7C) and was longest in the *TIAR*(+)+*LOXL1*‐*AS1*(−)+pre‐*miR*‐*374b*‐*5p* group. Finally, the number of VM tubes seen in vivo was lower in the *TIAR*(+), *LOXL1*‐*AS1*(−) and pre‐*miR*‐*374b*‐*5p* groups than that in their NCs and the least in the *TIAR*(+)+*LOXL1*‐*AS1*(−)+pre‐*miR*‐*374b*‐*5p* group (Figure 7D).

## 4 DISCUSSION

In this study, we showed that TIAR exerted anti‐tumour effects, while *LOXL1*‐*AS1* acted as an oncogene in glioma. TIAR could combine with *LOXL1*‐*AS1* and reduce its expression by lowering its stability. Moreover, TIAR overexpression and *LOXL1*‐*AS1* knockdown attenuated cell viability, migration, invasion and VM ability. However, *LOXL1*‐*AS1* overexpression reversed the anti‐cancer effects of TIAR overexpression in glioma cells. Our observations also showed that *miR*‐*374b*‐*5p* functions as a tumour suppressor in gliomas. Subsequently, we proved that *LOXL1*‐*AS1* was an RNA sponge for *miR*‐*374b*‐*5p* and downregulated its expression. Additionally, MMP14 acts as an oncogene in gliomas, while *miR*‐*374b*‐*5p* could mediate MMP14 expression by binding to their 3′UTRs, thus attenuating the malignant cell behaviours and VM in glioma. Finally, the concerted action of overexpressed TIAR, silenced *LOXL1*‐*AS1* and overexpressed *miR*‐*374b*‐*5p* repressed tumour growth as well as VM in vivo and prolonged the survival of nude mice.

RBPs play key functions in tumorigenesis and development at splicing, transcriptional, translational, intracellular‐transport and modification levels. The RBP *NONO* plays an oncogenic role in breast cancer and modifies *SKP2* and *E2F8* in the post‐transcriptional phase.^27^ *SORBS2* enhanced the stability of *WFDC1* and *IL*‐*17D* and inhibited the invasion in ovarian cancer.^28^ A recent study suggested that the lncRNA *MT1JP* communicating with TIAR post‐transcriptionally regulates P53 in tumours.^29^ In our study, TIAR was expressed at low levels in glioma. TIAR overexpression remarkably inhibited proliferation, metastasis and tube formation, while its knockdown tended to act in the opposite direction, confirming its tumour suppression role in glioma.

Malfunctions of lncRNAs play a unique role in oncogenesis and progression. A recent study identified the promotion of *lnc_000231* in cervical cancer, which acts by interacting with *miR*‐*497*‐*5p*.^30^ The lncRNA *ATB* binds to *EZH2* and downregulates the expression of DAB2IP, CDH1, LATS2, FOXC1 and CDX1, thus facilitating the progression of ovarian cancer.^31^ Furthermore, our study showed that *LOXL1*‐*AS1* is highly expressed in glioma tissues and cells, and its downregulation repressed the proliferation, metastasis and VM ability of these cells. These results confirmed that LOXL1‐AS1 has an aggressive role in glioma. Silencing *LOXL1*‐*AS1* suppresses cell proliferation in glioblastoma,^32^ which is consistent with our findings. Moreover, *LOXL1*‐*AS1* enhances the proliferation and invasion in medulloblastoma^33^ and negatively modulates *miR*‐*3128*, resulting in attenuation of the malignancy of H1299 and A549 lung cancer cells.^34^ However, the relationship between TIAR and *LOXL1*‐*AS1* in regulating VM formation in glioma has not been explored.

Several studies have reported that RBPs can positively or negatively affect RNA stability, resulting in tumour growth. For example, LARP1 enhances the stability of *BCL2* but attenuates the stability of *BIK*, leading to the malignant progression of ovarian cancer.^35^ *Linc*‐*00313*, which is stabilized by UPF1, regulates *miR*‐*342*‐*3p* and *miR*‐*485*‐*5p*, eventually accelerating the progression of glioblastoma.^36^ In colorectal cancer, MBNL1 destabilizes *Snail* and inhibits the epithelial‑to‑mesenchymal transition and metastasis of tumour cells.^37^ In our study, *LOXL1*‐*AS1* expression was reduced in TIAR‐overexpressing glioma cells and increased in TIAR‐knockdown glioma cells. Moreover, the results showed that TIAR communicated with *LOXL1*‐*AS1* but had no effect on levels of the nascent transcript. It therefore decreased *LOXL1*‐*AS1* expression by reducing its half‐life. Co‐transfection with *TIAR* overexpression and *LOXL1*‐*AS1* knockdown plasmids greatly mediated the proliferation, migration, invasion and VM of glioma cells. Our results revealed that TIAR’s weakening of the stability of *LOXL1*‐*AS1* attenuated the progression of glioma. Moreover, overexpression of *LOXL1*‐*AS1* reversed the anti‐tumour effects of TIAR overexpression in glioma cells. The function of *LOXL1*‐*AS1* in promoting adverse biological behaviours and VM in glioma has not been previously established.

Growing evidence has focused on the role of the relationship between lncRNAs and miRNAs in the mechanism of tumorigenesis. For example, *LINC*‐*PINT* modified *miR*‐*767*‐*5p*/*TET2* and inhibited malignant behaviour in thyroid cancer.^38^ *lnc*‐*NEAT1* acts as an oncogene to sponge *miR*‐*486*‐*5p*, and its suppression downregulates the malignant progression of colorectal cancer.^39^ The lncRNA *EBLN3P*, functioning as a competitive sponge of *miRNA*‐*144*‐*3p*, positively modulates DOCK4 in the ceRNA pathway and facilitates adverse processes in liver cancer.^40^ To confirm the function of *LOXL1*‐*AS1* in glioma, we used Starbase and predicted *miR*‐*374b*‐*5p* as a target of *LOXL1*‐*AS1*. A dual‐luciferase reporter assay was performed to assess whether *LOXL1*‐*AS1* binds to *miR*‐*374b*‐*5p*. *LOXL1*‐*AS1* upregulation suppressed *miR*‐*374b*‐*5p* expression, while *miR*‐*374b*‐*5p* overexpression reduced *LOXL1*‐*AS1* expression. Moreover, *miR*‐*374b*‐*5p* downregulation reduced the anti‐tumour effects of sh‐*LOXL1*‐*AS1* in glioma. These results indicate that *LOXL1*‐*AS1* exerts sponge‐like effect on *miR*‐*374b*‐*5p*, thereby regulating malignant cellular behaviours and VM in glioma. Consistent with our reports, *LOXL1*‐*AS1* sponges *miR*‐*541*‐*3p* by interacting with *CCND1*, thereby driving cell cycle progression and proliferation in prostate cancer.^41^ *LOXL1*‐*AS1* facilitates the adverse processes of gastric cancer by modulating *miR*‐*142*‐*5p* in a sponge way.^42^ Additionally, *LOXL1*‐*AS1* could regulate *miR*‐*324*‐*3p* like a sponge and accelerate the pernicious processes of cholangiocarcinoma and non‐small‐cell lung cancer.^43^, ^44^

Herein, we established an implanted tumour model in nude mice and observed the minimum tumour volume and longest survival in the TIAR(+)+*LOXL1*‐*AS1*(−)+pre‐*miR*‐*374b*‐*5p* group. Furthermore, dual staining for CD34 and PAS demonstrated that VM tubes were the fewest in the co‐transfected group. To date, it has been established that TIAR combined with *LOXL1*‐*AS1* can regulate *miR*‐*374b*‐*5p*; however, the mechanism by which *miR*‐*374b*‐*5p* regulates VM formation in glioma has not been proven.

miRNA dysfunction is found in most tumour cellular processes. For instance, *miR*‐*3666* targets *STAT3* and modulates the activity of *AK4*, exhibiting a suppressive role in ovarian cancer.^45^ *miRNA*‐*506*‐*3p* targets *EZH2* and negatively modulates the expression of *β*‐*catenin* in serous ovarian cancer.^46^ In this study, *miR*‐*374b*‐*5p* was downregulated in glioma, which revealed its function as a tumour suppressor. Similarly, *miR*‐*374b*‐*5p* targets *FOXP1* and exerts a protective role in non–small‐cell lung cancer and ovarian cancer.^47^, ^48^ Additionally, both *miR*‐*374b*‐*5p* and *miR*‐*454*‐*3p* exert inhibitory effects by regulating *ZEB2* in bladder cancer.^49^ Moreover, *miR*‐*374b* targets *FOXM1*, and its overexpression mediates adverse effects in cervical cancer.^50^ We also proved that MMP14 promotes malignant cell behaviours in gliomas. Likewise, MMP14 is highly expressed in gliomas where it acts as a mediator of migration.^51^ We also noticed that *miR*‐*374b*‐*5p* restrained MMP14 by binding to its 3′UTRs according to the dual‐luciferase reporter assay and Western blot, leading to the inhibition of VM by glioma cells. Similarly, MMP2 and MMP9 function as the key mediator of VM in glioma, while the suppression of Tenascin‐c attenuates AKT phosphorylation, and downregulates MMP2 and MMP9 expression, thus repressing VM in gliomas.^52^ Additionally, the upregulation of MMP14 reduced the inhibitory effects of overexpressed *miR*‐*374b*‐*5p* in glioma cells.

Overall, our study shows for the first time that TIAR plays a tumour‐suppressive role, while *LOXL1*‐*AS1* plays an aggressive role in glioma. TIAR downregulates *LOXL1*‐*AS1* by reducing its stability. *LOXL1*‐*AS1* overexpression reversed the anti‐tumour effects of TIAR overexpression in glioma cells. Moreover, *LOXL1*‐*AS1* knockdown upregulated *miR*‐*374b*‐*5p*, leading to the downregulation of MMP14, eventually suppressing malignant cell behaviour and VM in glioma cells. Our study shows that the TIAR/*LOXL1*‐*AS1*/*miR*‐*374b*‐*5p*/MMP14 axis has significant effects in regulating VM in glioma, which could help reveal novel targets for glioma therapy.

## CONFLICTS OF INTEREST

The authors declare that they have no competing interests.

## AUTHOR CONTRIBUTIONS

**Bolong Yi:** Data curation (equal); Funding acquisition (equal); Investigation (lead); Writing – original draft (lead); Writing – review & editing (equal). **Hao Li:** Funding acquisition (equal); Investigation (equal); Methodology (equal); Software (equal). **Heng Cai:** Data curation (equal); Methodology (equal). **Xin Lou:** Formal analysis (equal); Investigation (equal); Software (equal); Writing – review & editing (equal). **Mingjun Yu:** Formal analysis (equal); Software (equal). **Zhen Li:** Data curation (equal); Funding acquisition (lead); Supervision (lead); Writing – review & editing (equal).

## ETHICAL APPROVAL

Written informed consent was signed voluntarily by all patients. This research is supported by the Shengjing Hospital Ethical Committee (approval ID: 2020PS012K) and the panel of experts on Laboratory Animal Care of the Shengjing Hospital (approval ID: 2020PS015K).

## Supporting information

Fig S1

Fig S2

Fig S3

Table S1

## Data Availability

We confirm that we will share the data underlying the findings reported in this manuscript and allow researchers to verify the results presented, replicate the analysis, and conduct secondary analyses.
