# Supplementary figures and images for: LOXL1‐AS1 communicating with TIAR modulates vasculogenic mimicry in glioma via regulation of the miR‐374b‐5p/MMP14 axis
Source: J Cell Mol Med. 2021 Dec 8;26(2):475–90. doi: 10.1111/jcmm.17106 (PMC8743654; doi:10.1111/jcmm.17106)

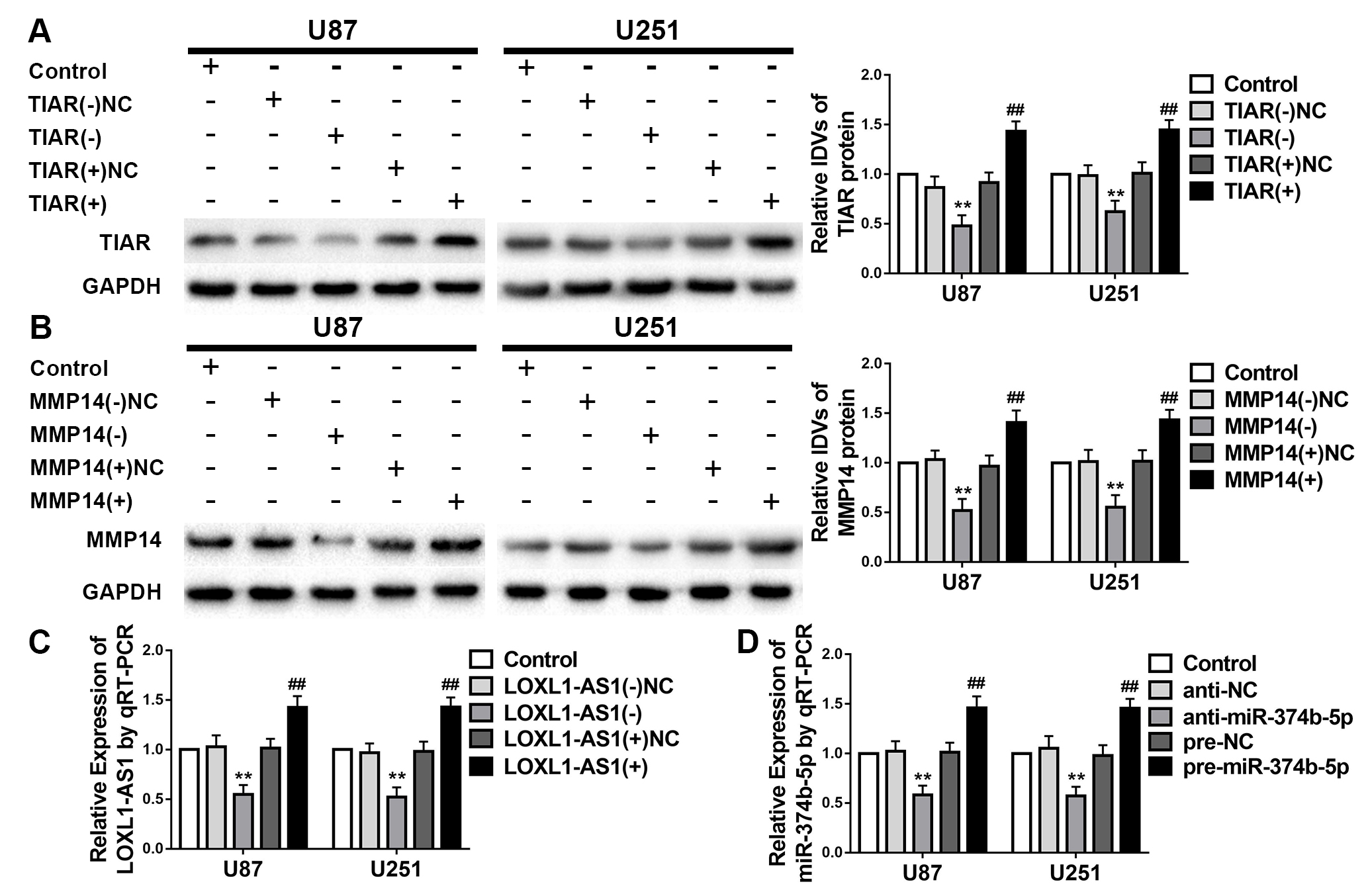

Supplement: Supplementary file 1 — Fig S1 [file JCMM-26-475-s002.jpg]

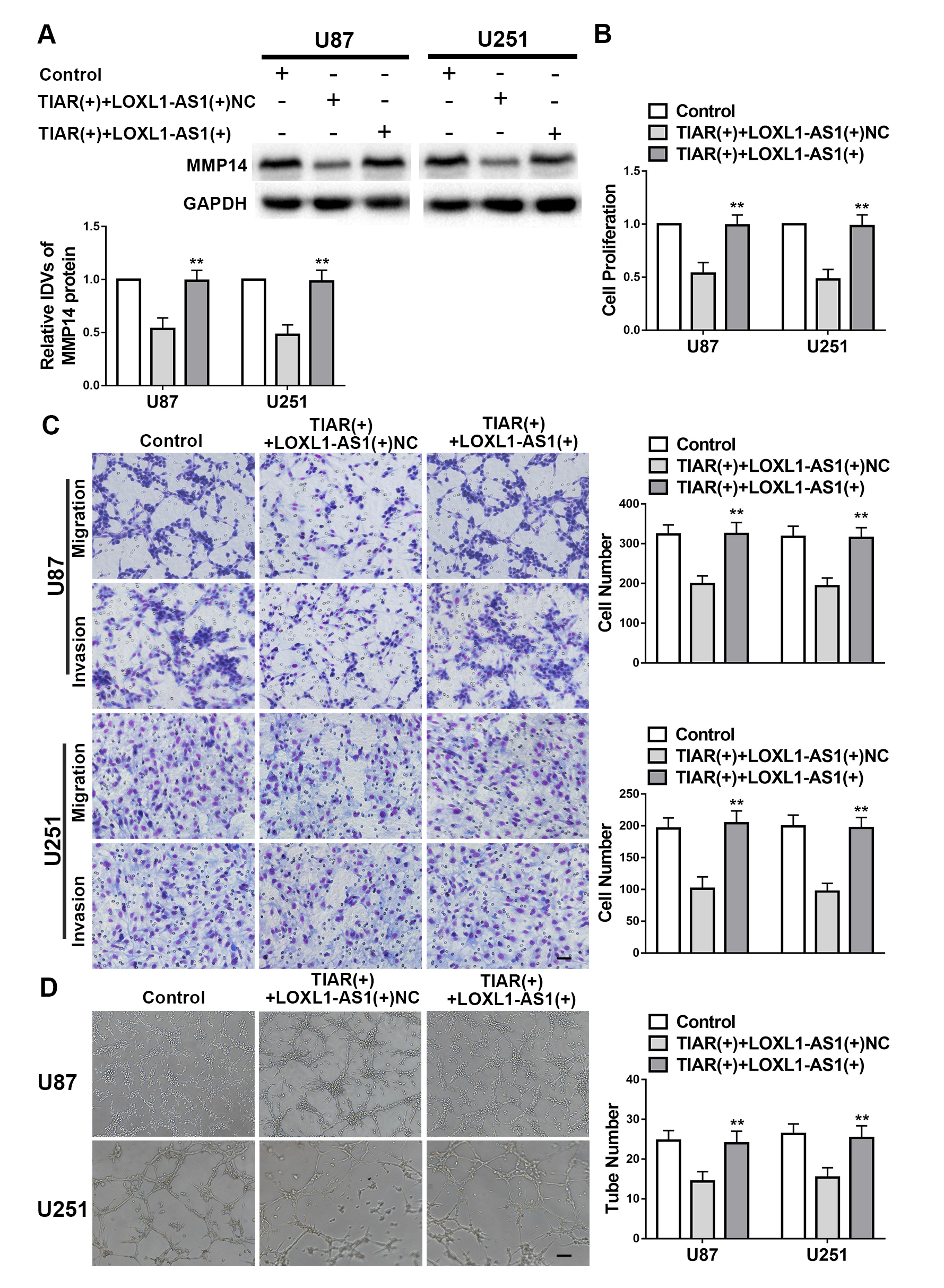

Supplement: Supplementary file 2 — Fig S2 [file JCMM-26-475-s004.jpg]

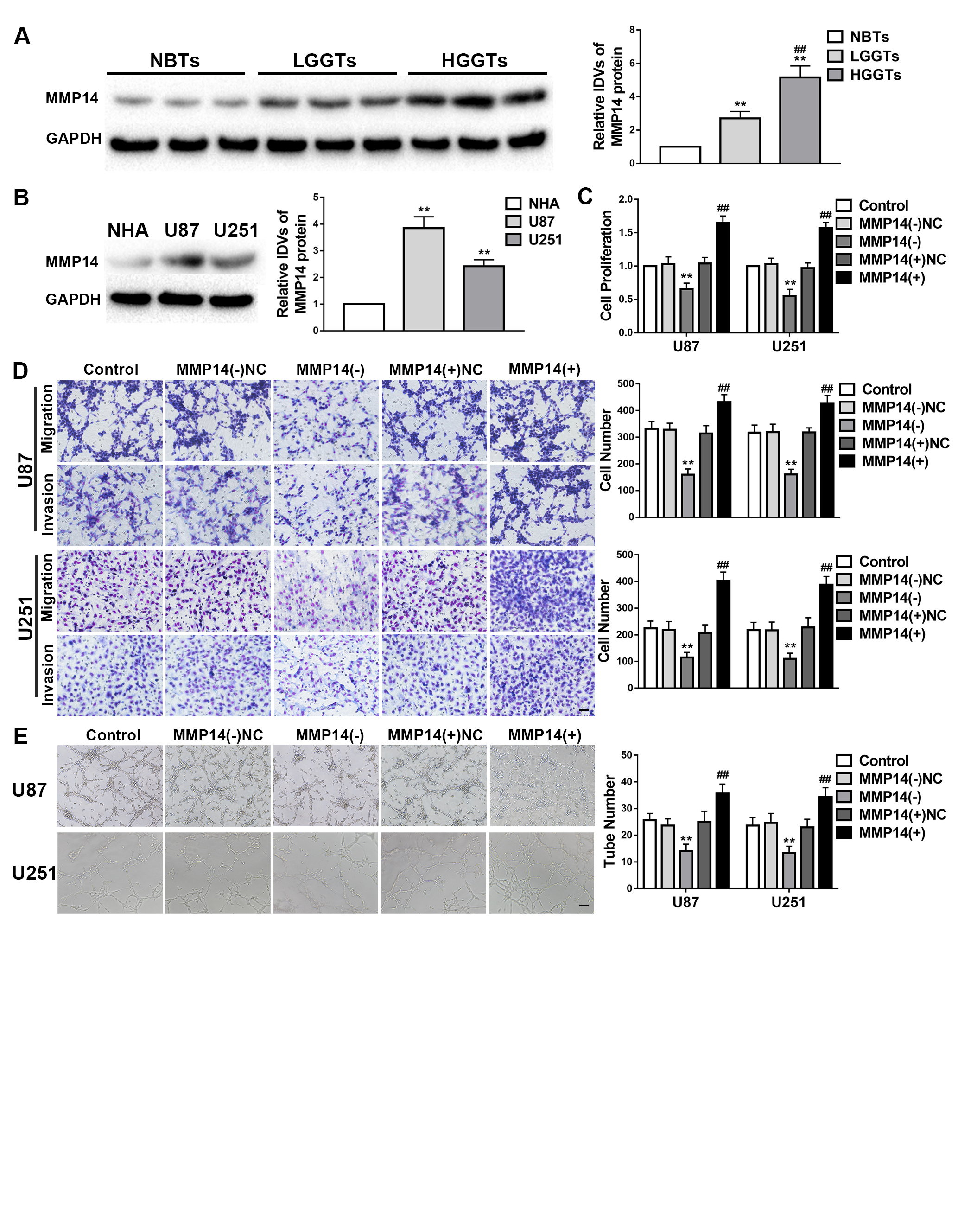

Supplement: Supplementary file 3 — Fig S3 [file JCMM-26-475-s003.jpg]
