# Supplementary material for: LOXL1‐AS1 communicating with TIAR modulates vasculogenic mimicry in glioma via regulation of the miR‐374b‐5p/MMP14 axis
Source: J Cell Mol Med. 2021 Dec 8;26(2):475–90. doi: 10.1111/jcmm.17106 (PMC8743654; doi:10.1111/jcmm.17106)
Supplement: Supplementary file 4 — Table S1 [file JCMM-26-475-s001.docx]

**Table S1. The primers for TIAR, LOXL1-AS1, miR-374b-5p, MMP14, GAPDH, and U6 in quantitative real-time PCR.**

| Primers | Forward | Reverse |
| --- | --- | --- |
| TIAR | 5'-ACAGACATTCTCACCATTTGGAC-3' | 5'-GTAGTACCGTTCACCGAAACAAT-3' |
| LOXL1-AS1 | 5'-GGCTCCTGTCTCTACCTCCACTTC-3' | 5'-AATCGGCTCCTGTCTCCACTGG-3' |
| MMP14 | 5'-CAAGATTGATGCTGCTCTCTTC-3' | 5'-ACTTTGATGTTCTTGGGGTACT-3' |
| GAPDH | 5'-AAATCCCATCACCATCTTCCAG-3' | 5'-TGATGACCCTTTTGGCTCCC-3' |
| miR-374b-5p | 5'- GCGCGATATAATACAACCTGC -3' | 5'- AGTGCAGGGTCCGAGGTATT -3' |
| U6 | 5'- AGAGAAGATTAGCATGGCCCCTG -3' | 5'-ATCCAGTGCAGGGTCCGAGG-3' |
|  | | |
| Primers | RT Primer |  |
| miR-374-5p | 5'-GTCGTATCCAGTGCAGGGTCCGAGGT  ATTCGCACTGGATACGACCACTTA-3' |  |
| U6 | 5'-GTCGTATCCAGTGCAGGGTCCGAGGT  ATTCGCACTGGATACGACAAAATA-3' |  |
